# Supplementary material for: Transcriptomic Insights into Mechanisms of Early Seed Maturation in the Garden Pea (Pisum sativum L.)
Source: Cells. 2020 Mar 23;9(3):779. doi: 10.3390/cells9030779 (PMC7140803; doi:10.3390/cells9030779)

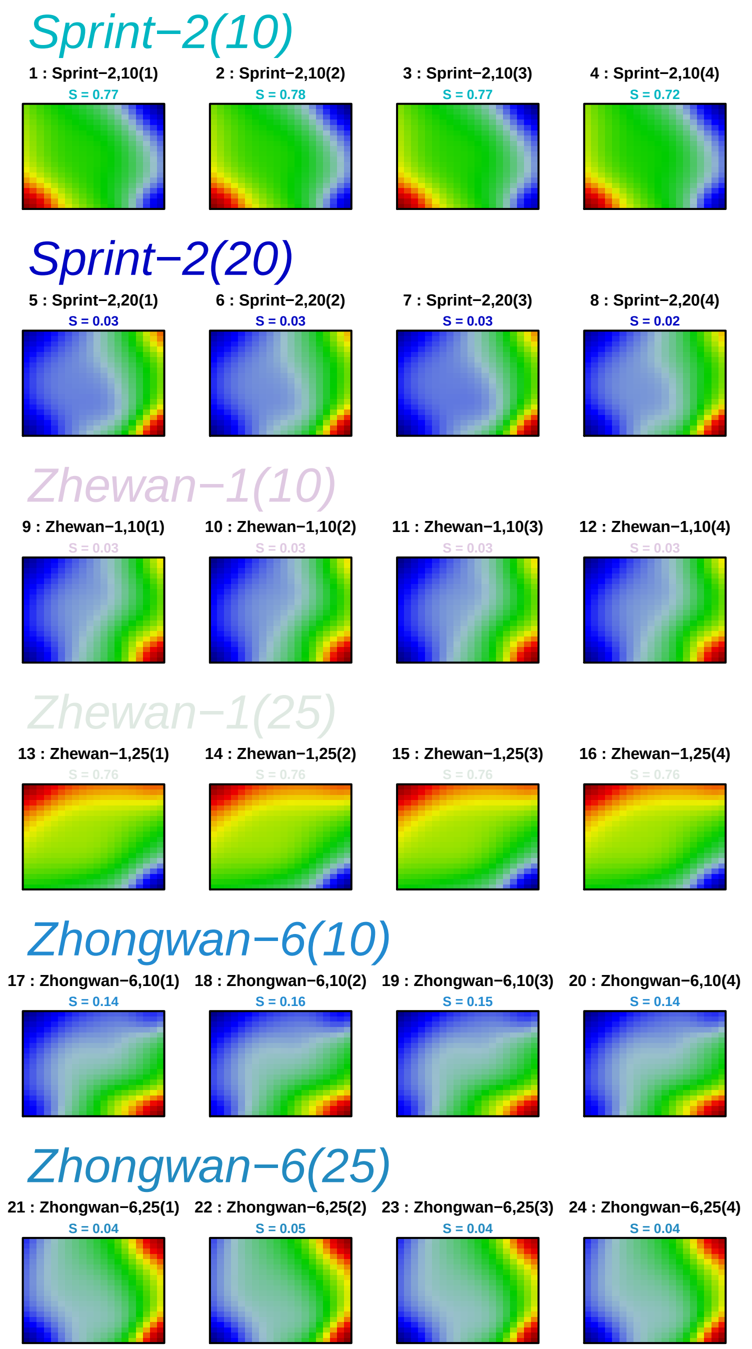
Sprint−2(10)
1 : Sprint−2,10(1)
S = 0.77
2 : Sprint−2,10(2)
S = 0.78
3 : Sprint−2,10(3)
S = 0.77
4 : Sprint−2,10(4)
S = 0.72
Sprint−2(20)
5 : Sprint−2,20(1)
S = 0.03
6 : Sprint−2,20(2)
S = 0.03
7 : Sprint−2,20(3)
S = 0.03
8 : Sprint−2,20(4)
S = 0.02
Zhewan−1(10)
9 : Zhewan−1,10(1)
S = 0.03
10 : Zhewan−1,10(2)
S = 0.03
11 : Zhewan−1,10(3)
S = 0.03
12 : Zhewan−1,10(4)
S = 0.03
Zhewan−1(25)
13 : Zhewan−1,25(1)
S = 0.76
14 : Zhewan−1,25(2)
S = 0.76
15 : Zhewan−1,25(3)
S = 0.76
16 : Zhewan−1,25(4)
S = 0.76
Zhongwan−6(10)
17 : Zhongwan−6,10(1)
S = 0.14
18 : Zhongwan−6,10(2)
S = 0.16
19 : Zhongwan−6,10(3)
S = 0.15
20 : Zhongwan−6,10(4)
S = 0.14
Zhongwan−6(25)
21 : Zhongwan−6,25(1)
S = 0.04
22 : Zhongwan−6,25(2)
S = 0.05
23 : Zhongwan−6,25(3)
S = 0.04
24 : Zhongwan−6,25(4)
S = 0.04

Sprint−2(10)

1 : Sprint−2,10(1) 2 : Sprint−2,10(2) 3 : Sprint−2,10(3) 4 : Sprint−2,10(4)

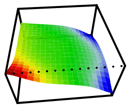 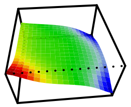 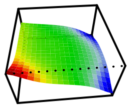 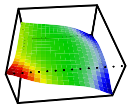

Sprint−2(20)

5 : Sprint−2,20(1) 6 : Sprint−2,20(2) 7 : Sprint−2,20(3) 8 : Sprint−2,20(4)

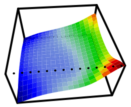 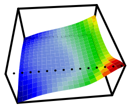 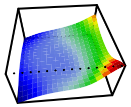 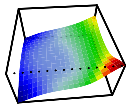

Zhewan−1(10)

9 : Zhewan−1,10(1) 10 : Zhewan−1,10(2) 11 : Zhewan−1,10(3) 12 : Zhewan−1,10(4)

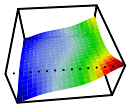 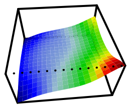 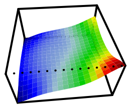 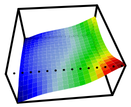

Zhewan−1(25)

13 : Zhewan−1,25(1) 14 : Zhewan−1,25(2) 15 : Zhewan−1,25(3) 16 : Zhewan−1,25(4)

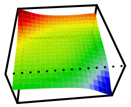 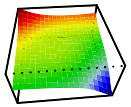 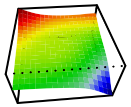 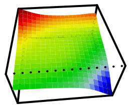

Zhongwan−6(10)

17 : Zhongwan−6,10(1) 18 : Zhongwan−6,10(2) 19 : Zhongwan−6,10(3) 20 : Zhongwan−6,10(4)

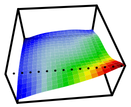 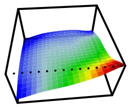 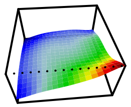 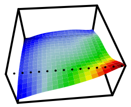

Zhongwan−6(25)

21 : Zhongwan−6,25(1) 22 : Zhongwan−6,25(2) 23 : Zhongwan−6,25(3) 24 : Zhongwan−6,25(4)

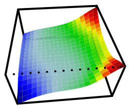 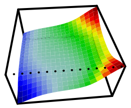 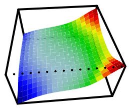 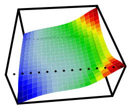

Supplement: Supplementary file 1 [file cells-09-00779-s001.zip › Supplementary materials/FileS3.zip!/FileS2/Hormones - Results/Expression Portraits.pdf]
